# Comparison of Finite Difference and Finite Volume Simulations for a Sc-Drying Mass Transport Model

**DOI:** 10.3390/gels6040045

**Published:** 2020-11-25

**Authors:** Ilka Selmer, Patricio Farrell, Irina Smirnova, Pavel Gurikov

**Affiliations:** 1 Institute for Thermal Separation Processes, Hamburg University of Technology, Eißendorfer Straße 38, 21073 Hamburg, Germany; irina.smirnova@tuhh.de; 2 Weierstrass Institute (WIAS), Mohrenstr. 39, 10117 Berlin, Germany; patricio.farrell@wias-berlin.de; 3 Laboratory for Development and Modelling of Novel Nanoporous Materials, Hamburg University of Technology, Eißendorfer Straße 38, 21073 Hamburg, Germany

**Keywords:** aerogel particles, supercritical drying, finite difference method, finite volume method, mass transport simulation, advection-diffusion equation

## Abstract

Different numerical solutions of a previously developed mass transport model for supercritical drying of aerogel particles in a packed bed [Part 1: Selmer et al. 2018, Part 2: Selmer et al. 2019] are compared. Two finite difference discretizations and a finite volume method were used. The finite volume method showed a higher overall accuracy, in the form of lower overall Euclidean norm ($l_{2}$) and maximum norm ($l_{\infty }$) errors, as well as lower mole balance errors compared to the finite difference methods. Additionally, the finite volume method was more efficient when the condition numbers of the linear systems to be solved were considered. In case of fine grids, the computation time of the finite difference methods was slightly faster but for 16 or fewer nodes the finite volume method was superior. Overall, the finite volume method is preferable for the numerical solution of the described drying model for aerogel particles in a packed bed.

## 1. Introduction

Supercritical drying of wet gels, such that they become porous aerogels, is the crucial step in aerogel production. The drying process uses supercritical CO_2_ as extraction medium and is; thus, conducted under moderate temperature but elevated pressure and; therefore, may be cost-intensive. A comprehensive overview of experimental studies and mass transport models investigating the kinetics of supercritical drying of gels is given by Şahin et al. [1]. Within the last two years, a several model of the supercritical drying for spherical gel particles were developed [2,3,4,5,6,7]. All these works show that the gel drying process for particles is significantly faster than for monoliths of comparable size [2,3,4,5,6,7]. This observation highlights the fact that aerogel particles are highly promising for future applications in industrial products. Selmer et al. developed their physical mass transport model for the supercritical drying of gel particles in a packed bed and analyzed mass transfer steps that limit the overall drying kinetic to optimize the process [2,3]. Hatami et al. developed a similar model and improved the optimizing procedure to allow a fast drying process with low CO_2_ consumption [6].

All reported mass transport models result in a set of coupled partial differential equations if the mass transport in the outer bulk fluid is considered additionally to the mass transport within the gel particles [3,4,5,6]. Şahin et al. and Hatami et al. discretized the spatial dimensions via the finite difference method and solved the resulting coupled set of differential-algebraic equations using the built-in ordinary differential equation solver in Matlab [4,5,6]. Selmer et al. discretized the spatial dimension of the gel particles via the finite difference method [8,9] and the spatial dimension of the bulk fluid in the autoclave via the finite volume method [3,10]. The finite volume method can preserve discretely important physical quantities such as mass [11].

During the supercritical drying process, the physical properties of supercritical CO_2_ and its mixture with ethanol or other solvents strongly depend on the composition of the mixture which varies during the drying process from pure ethanol (or another solvent) in the gel to almost pure CO_2_ [3]. Therefore, especially in mass transport models describing the supercritical drying process of small particles, a high spatial resolution of the domain with gel particles is required for accurate results. This, in turn, leads to high computation times.

For this reason, we set the aim of this work so as to investigate the behavior of numerical solutions of gel particles drying models according to their accuracy and efficiency, to increase the confidence in the simulation results reported so far.

In the present paper, two possible numerical discretization techniques of the resulting partial differential equations of Selmer et al.’s model [2,3] are implemented, namely the finite difference method [8,9] and the finite volume method [10]. Both discretization techniques are compared numerically by studying the quality of the approximations via Euclidean norm ($l_{2}$) and maximum norm ($l_{\infty }$) errors, as well as the efficiency via the condition numbers of the linear systems to be solved.

The supercritical drying of spherical, ethanol-filled silica gels packed in a cylindrical bed serves as the example system. Two initial ethanol concentrations in the bulk fluid, which represent the pressurization using CO_2_ with or without addition of excess solvent to the wet gels, are evaluated. Both pressurization techniques are applicable and implemented in supercritical drying processes.

## 2. Mass Transport Model of Supercritical Drying

**Figure 1:**
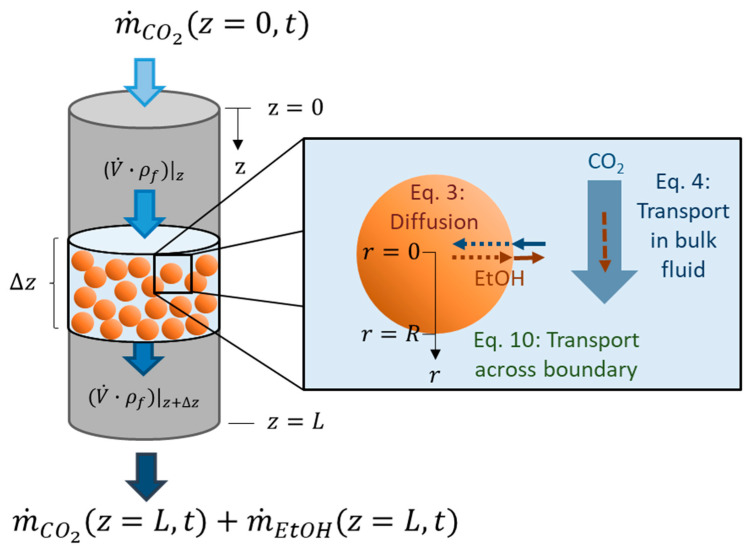
Supercritical drying of gel particles in a packed bed (*z*—axial coordinate of packed bed, *z* = 0—top of packed bed, *z* = *L*—bottom of packed bed, Δz—slice of packed bed, ${\dot{m}}_{CO_{2}}$—CO_2_ flow,$\;{\dot{m}}_{EtOH}$—ethanol flow, $\dot{V}$—volume flow, $\rho_{f}$—fluid density (mixture of CO_2_ and ethanol), *r*—radial coordinate of spherical gel particle, *r* = 0—gel particle center, *r* = *R*—gel particle radius, *t*—time).

The aim of supercritical drying is to extract a solute (in our case ethanol) from a wet gel using supercritical CO_2_ (component 1). The mass transport model presented here, and discussed in Selmer et al. 2018 from the physical point of view [2], results in a system of coupled partial differential equations. The partial differential equations are written here in terms of concentration of ethanol (component 2), $c_{2}$. In the drying process, ethanol is transported by diffusion from the inside of the porous gel particles (Equation (1)) to the bulk fluid (Figure 1). The flow of the bulk fluid through the packed bed is modeled by convection (Equation (2)) (Figure 1). The packed bed leads to non-ideal flow behavior, which is included through a time dependent dispersion factor $D_{L}$ (Equation (2)). Thus, two domains are considered: The spherical gel particles (subscript g), described by radial mass transport along the radial axis r; and the bulk fluid (subscript f), described by axial mass transport along the axial axis z. Both domains are connected due to the mass transfer of ethanol across the boundary. This connection appears as a boundary condition for the mass transport within the particle (Equation (8)) and as a source term $source_{2,f}$ for the mass transport of the bulk fluid (Equation (2)). It is assumed in our model that the gel particles are monodisperse and evenly distributed along the cylindrical autoclave. Since the mass transport within the gel particles depends on the mass transport of the bulk fluid, which in its turn varies along the axial packed bed height z, the mass transport within the gel particles should also depend on their position z in the packed bed. This fact is explicitly stated in (Equation (1)). The drying process is a dynamic process and thus depends on the time denoted with t. The source term $source_{2,f}$ describes the ethanol being extracted from the gel particles (at axial position z in the packed bed) into the bulk fluid. The term is calculated as the temporal change of the amount of ethanol (in moles) within all gel particles divided by the accessible volume of the bulk fluid $V_{f}=A_{ac}\cdot \psi \cdot L$ (Equation (4)). $A_{ac}$ stands for the cross-sectional area of the autoclave, ψ for the porosity of the packed bed and L for the length of the packed bed. The temporal change of the ethanol amount within the porous gel sphere is negative during the course of the drying since ethanol concentration with time decreases, but the source term itself is positive.

Physical properties of supercritical CO_2_ and its mixture with ethanol or other solvents strongly depend on the system parameters such as temperature, pressure and composition of the mixture [2]. The temperature and pressure are treated as constant during the drying process. The composition of binary mixture $x_{2}$ is directly related to the mixture concentration c and ethanol concentration $c_{2}$ (Equation (5)) and varies during the drying from pure ethanol ($x_{2}=1$) to pure CO_2_ ($x_{2}=0$). Physical properties, such as the effective diffusion coefficient $D_{g}$, the mixture concentration $c_{g}$ and the fluid density $\rho_{f,}$ as well as the parameters that are functions thereof, such as the interstitial fluid velocity u (Equation (3)) and the mass transfer coefficient β (Equation (8)), are highly dependent on the radial, axial and temporal distribution of the mixture compositions in the gel body $x_{2,g}\left(r,z,t\right)$ and the bulk fluid $x_{2,f}\left(z,t\right)$. For example, at a pressure of 10 MPa and a temperature of 321 K, the parameters being directly dependent from the mixture composition can vary during supercritical drying between

$1.41\cdot 10^{-9}$ and $7.68\cdot 10^{-9}\frac{m^{2}}{s}$ for the effective diffusion coefficient $D_{g}$ (assumption: Gel porosity $\varepsilon_{g}\;=0.93$, gel tortuosity $\tau_{g}\;=3.48$),
$9.67\cdot 10^{3}$ and $1.77\cdot 10^{4}\frac{mol}{m^{3}}$ for the mixture concentration $c_{g}$ and the fluid density $\rho_{f}$,
$5.54\cdot 10^{-9}$ and $2.87\cdot 10^{-8}\frac{m^{2}}{s}$ for the diffusion coefficient in the bulk fluid,
$3.09\cdot 10^{-5}$ and $7.18\cdot 10^{-4}\;Pa\cdot s$ for the viscosity of the bulk fluid and between
2.34 and $1.66\cdot 10^{2}$ for the resulting Schmidt number.

Parameters that are independent from the mixture composition are written in bold font throughout the text and as follows: The surface of a single spherical gel particle $A_{p}$, the number of particles in the packed bed $N_{p}$, the cross-sectional area of the autoclave $A_{ac}$, the length of the packed bed L, the porosity of the packed bed ψ and the mass flow rate of the ethanol–CO_2_ mixture $\dot{m}$.

(1) $$\;\frac{\partial c_{2,g}\left(r,z,t\right)}{\partial t}=\frac{1}{r^{2}}\;\left[\;\frac{\partial }{\partial r}\left(r^{2}\cdot D_{g}\left(x_{2,g}\left(r,z,t\right)\right)\;\cdot c_{g}\left(x_{2,g}\left(r,z,t\right)\right)\cdot \frac{\partial x_{2,g}\left(r,z,t\right)}{\partial r}\right)\right]\;$$

(2) $$\frac{\partial c_{2,f}\left(z,t\right)}{\partial t}=\;D_{L}\left(t\right)\cdot \frac{\partial^{2}c_{2,f}\left(z,t\right)}{\partial z^{2}}-\frac{\partial \left(c_{2,f}\left(z,t\right)\cdot u\left(x_{2,f}\left(z,t\right)\right)\right)}{\partial z}\;+source_{2,f}\left(x_{2,g}\left(r,z,t\right),x_{2,f}\left(z,t\right)\right)\;$$

(3) $$\;u\left(x_{2,f}\left(z,t\right)\right)=\frac{\dot{m}}{A_{ac}\cdot \psi \cdot \rho_{f}\left(x_{2,f}\left(z,t\right)\right)}$$

(4) $$source_{2,f}\left(x_{2,g}\left(r,z,t\right),x_{2,f}\left(z,t\right)\right)=-\frac{N_{p}}{A_{ac}\cdot \psi \cdot L}\cdot \frac{\partial }{\partial t}\left(\overset{r=R}{\underset{r=0}{\int }}4\cdot \pi \cdot r^{2}\cdot \varepsilon_{g}\cdot c_{2,g}\left(r,z,t\right)\;dr\right)\;$$

(5) $$\;c_{2}=x_{2}\cdot c\;$$

Initial and boundary conditions are presented in Equations (6)–(8) for the gel particle domain and in Equations (10)–(12) for the bulk fluid domain. For each gel sphere (at a certain packed bed height z), the boundary conditions were given by the Neumann condition at the center (Equation (7)) and by the molar flux across the boundary layer at the surface (Equation (8)).

(6) $$\begin{array}{cc} \forall r,\forall z,t=0 & c_{2,g}\left(r,z,0\right)=c_{2,\;g,0} \end{array}$$

(7) $$\begin{array}{cc} \;r=0,\forall z,\;\forall t & \frac{\partial c_{2,g}\left(0,z,t\right)}{\partial r}=0 \end{array}$$

(8) $$\begin{array}{c} r=R,\\ \forall z,\forall t \end{array}\;\begin{array}{cc} -D_{g}\left(x_{2,g}\left(R,z,t\right)\right) & \cdot \frac{\partial c_{2,g}\left(R,z,t\right)}{\partial r}\\ & =\beta \left(x_{2,f}\left(z,t\right),u(x_{2,f}\left(z,t\right)\right)\cdot \left(c_{2,g}\left(R,z,t\right)-c_{2,\;f}\left(z,t\right)\right) \end{array}$$

For the top of the packed bed, the Danckwerts’ boundary condition [12] was chosen so that no loss of ethanol across the upper boundary occurs due to dispersion of ethanol (Equation (11)). It was useful to define the flux of ethanol $f_{2}$ as follows:

(9) $$\;f_{2}:=-D_{L}\cdot \frac{\partial c_{2,f}}{\partial z}+u\cdot c_{2,f}.\;$$

In order to simplify the boundary condition at the top of the packed bed: The flux of ethanol across the upper boundary is set to zero (Equation (11)) according to the Danckwerts’ boundary condition [12].

At the bottom of the packed bed, a Neumann boundary condition was used (Equation (12)).

(10) $$\begin{array}{cc} \forall z,\;t=0 & c_{2,f}\left(z,0\right)=c_{2,\;f,0} \end{array}$$

(11) $$\begin{array}{cc} z=0,\;\forall t & 0=\;u\left(x_{2,f}\left(0,t\right)\right)\cdot c_{2,\;f}\left(0,t\right)-D_{L}\left(t\right)\cdot \frac{\partial c_{2,f}\left(0,t\right)}{\partial z}=f_{2}\left(0,t\right) \end{array}$$

(12) $$\begin{array}{cc} z=L,\;\forall t & \frac{\partial c_{2,f}\left(L,t\right)}{\partial z}=0 \end{array}$$

## 3. Numerical Solution of the Mass Transport Model

The coupled partial differential equations were solved numerically. Equation (1), which describes the mass transport within the porous gel particles, was always solved using the finite difference method (FDM), whereas Equation (2), which describes the mass transport of the bulk fluid in the packed bed, was solved using either the FDM (Section 3.1) or the finite volume method (FVM) (Section 3.2), (Supplementary Materials).

Equation (1) was discretized using an explicit scheme, since it is easy to implement. Without its dependencies on r, z , t, Equation (1) is written as follows:

(13) $$\begin{array}{cc} \forall z & \frac{\partial c_{2,g}}{\partial t}=\frac{1}{r^{2}}\;\left[\;\frac{\partial }{\partial r}\left(r^{2}c_{g}D_{g}\frac{\partial x_{2,g}}{\partial r}\right)\right] \end{array}$$

which gives, after applying the product rule,

(14) $$\begin{array}{cc} \forall z & \frac{\partial c_{2,g}}{\partial t}=\frac{2c_{g}D_{g}}{r}\frac{\partial x_{2,g}}{\partial r}+D_{g}\frac{\partial c_{g}}{\partial r}\;\frac{\partial x_{2,g}}{\partial r}+c_{g}\frac{\partial D_{g}}{\partial r}\;\frac{\partial x_{2,g}}{\partial r}+c_{g}D_{g}\frac{\partial^{2}x_{2,g}}{\partial r^{2}}. \end{array}$$

**Figure 2:**
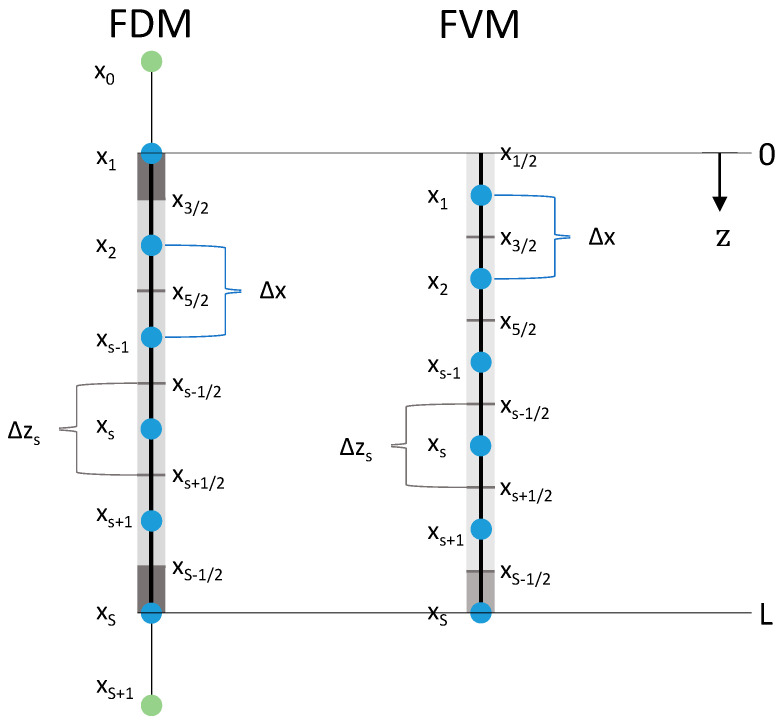
Axial discretizations of the packed bed for FDM and FVM. S—number of nodes, $x_{s}$—s^th^ node (blue), $x_{0},x_{S+1}—$artificial node (green), Δx—distance between neighboring nodes, $x_{s+\frac{1}{2}}—$cell interface (in the middle of two neighboring nodes), $\Delta z_{s}—$length of volume element (grey boxes, boundary boxes in dark grey).

Equation (14) was discretized using a forward difference for the time derivative and central differences for the spatial derivatives

(15) $$\begin{array}{c} n=1,\ldots ,N\\ \forall s,\forall k \end{array}\;\begin{array}{cc} \frac{c_{2,g,n,s}^{k+1}-c_{2,g,n,s}^{k}}{\Delta t} & =\frac{2c_{g,n,s}^{k}D_{g,n,s}^{k}}{n\Delta r}\frac{x_{2,g,n+1,s}^{k}-x_{2,g,n-1,s}^{k}}{2\Delta r}\\ & +c_{g,n,s}^{k}\;\frac{D_{g,n+1,s}^{k}-D_{g,n-1,s}^{k}}{2\Delta r}\frac{x_{2,g,n+1,s}^{k}-x_{2,g,n-1,s}^{k}}{2\Delta r}\\ & +D_{g,n,s}^{k}\;\frac{c_{g,n+1,s}^{k}-c_{g,n-1,s}^{k}}{2\Delta r}\frac{x_{g,2,n+1,s}^{k}-x_{g,2,n-1,s}^{k}}{2\Delta r}\\ & +c_{g,n,s}^{k}D_{g,n,s}^{k}\frac{x_{2,g,n+1,s}^{k}-2x_{2,g,n,s}^{k}+x_{2,g,n-1,s}^{k}}{\Delta r^{2}} \end{array}$$

where the super index k refers to time, s to the axial index in the packed bed/autoclave domain (cf. Figure 2), and n to the radial index of the gel particle domain. N stands for the number of nodes used to discretize the gel particle domain. The temporal as well as both spatial discretizations were based on equidistant nodes resulting in constant time $\Delta t=t^{k}-t^{k-1}$ and constant radial steps $\Delta r=r_{n}-r_{n-1}\;$. We can rearrange Equation (15) to

(16) $$\begin{array}{c} n=1,\ldots ,N\\ \forall s,\forall k \end{array}\;\begin{array}{cc} c_{2,g,n,s}^{k+1}=c_{2,g,n,s}^{k} & +\frac{\Delta t}{\Delta r^{2}}\{[\left(1-\frac{1}{n}\right)x_{2,g,n-1,s}^{k}-2x_{2,g,n,s}^{k}\\ & +\left(1+\frac{1}{n}\right)x_{2,g,n+1,s}^{k}]D_{g,n,s}^{k}c_{g,n,s}^{k}\\ & +\frac{1}{4}[\left(x_{2,g,n-1,s}^{k}-x_{2,g,n+1,s}^{k}\right)(\left(D_{g,n-1,s}^{k}-D_{g,n+1,s}^{k}\right)c_{g,n,s}^{k}\\ & +\left(c_{g,n-1,s}^{k}-c_{g,n+1,s}^{k}\right)D_{g,n,s}^{k})]\}. \end{array}$$

The initial condition of the gel particle domain was set to

(17) $$\begin{array}{cc} \forall n,\forall s,\;k=1 & c_{2,g,n,s}^{0}=c_{2,\;g,start}. \end{array}$$

The boundary conditions of the gel particle domain were discretized using central differences for the spatial derivatives. Two artificial nodes (n=0, n=N+1) were placed beyond the boundaries n=1 and n=N and calculated as follows:

(18) $$\begin{array}{cc} n=0,\forall s,\;\forall k & c_{2,g,0,s}^{k}=c_{2,g,2,s}^{k},\;x_{2,g,0,s}^{k}=x_{2,g,2,s}^{k},\;c_{g,0,s}^{k}=c_{g,2,s}^{k},\;D_{g,0,s}^{k}=D_{g,2,s}^{k} \end{array}$$

(19) $$\begin{array}{cc} n=N+1,\forall s,\forall k & c_{2,g,N+1,s}^{k}=c_{2,g,N-1,s}^{k}-2\Delta r\frac{\beta_{s}^{k}}{D_{g,N,s}^{k}}\left(c_{2,g,N,s\;}^{k}-c_{2,f,s}^{k}\right). \end{array}$$

The artificial node n=0 was used to solve Equation (16) for $c_{2,g,1,s}^{k+1}$ and the artificial node n=N+1 for $c_{2,g,N,s}^{k+1}\;$.

The domain which describes the mass transport in the bulk fluid (Equation (2)) was solved with both numerical methods (Section 3.1 and Section 3.2). Without its dependencies on r, z, t, Equation (2) was written as follows:

(20) $$\frac{\partial c_{2,f}}{\partial t}=D_{L}\cdot \frac{\partial^{2}c_{2,f}}{\partial z^{2}}-\frac{\partial \left(c_{2,f}\cdot u\right)}{\partial z}+source_{2,f}.$$

Using this definition of the flux (Equation (9)) allows Equation (20) to be rewritten as

(21) $$\frac{\partial c_{2}}{\partial t}=-\frac{\partial f_{2}}{\partial z}+source_{2,f}.$$

The source term $source_{2,f}$ (Equation (4)), which describes the ethanol being extracted from porous gel particles to the bulk fluid in the packed bed with time, was discretized for both numerical methods in the same way (Equation (22)): It was calculated as the difference between the accumulated ethanol within a single porous gel particle (placed at axial position s ) at time t=k and at time t=k+1, divided by the time step $\Delta t=t^{k+1}-t^{k}$ and multiplied by the particles per bulk fluid volume $\frac{N_{p}}{V_{f}}=\frac{N_{p}}{A_{ac}\cdot \psi \cdot L}$ (Equation (22)). The accumulated ethanol within a single porous particle was calculated by the summation of the ethanol concentration $c_{2,g,n,s}^{k}$ in each spherical shell element n, times the volume of each spherical shell element $V_{g,n}$ times the gel porosity $\varepsilon_{g}$ (Equation (22)):

(22) $$\begin{array}{cc} \forall s,\forall k & source_{2,f,s}^{k}=\frac{N_{p}}{A_{ac}\cdot \psi \cdot L\cdot \Delta t}\cdot \left(\overset{N}{\underset{n=1}{\sum }}c_{2,g,n,s}^{k}\cdot V_{g,n}\cdot \varepsilon_{g}-\overset{N}{\underset{n=1}{\sum }}c_{2,g,n,s}^{k+1}\cdot V_{g,n}\cdot \varepsilon_{g}\right). \end{array}$$

### 3.1. Solving the Diffusion-Advection Equation Using the Finite Difference Method

Finite difference methods are a flexible tool to discretize partial differential equations and easy to implement.

Figure 2 shows the equidistant axial discretization of the bulk fluid/packed bed domain for the FDM and the FVM (Section 3.2). For FDM, the first discretization node was placed at z=0 and the last one at z=L. Therefore, both boundary volume elements/cells (grey boxes) were half of the length of the inner volume elements:

(23) $$\Delta z_{s}:=\{\begin{array}{c} \Delta z=\Delta x\\ \frac{\Delta z}{2}=\frac{\Delta x}{2} \end{array}\begin{array}{c} s=2,\ldots ,S-1\\ s=1,S\;. \end{array}$$

Additional to the number of nodes S in the packed bed domain, two more artificial nodes ($x_{0}$ and $x_{S+1}\;$) outside the domain were needed to discretize the boundary conditions via the FDMs.

In the following, we will distinguish between two versions of possible discretizations using the FDM to solve numerically the diffusion-advection equation. The difference is given by the discretization of the advective term in Equation (20).

#### 3.1.1. Discretization of Advection Term: Version A

In version A, the advective term $-\frac{\partial \left(c_{2,f}\cdot u\right)}{\partial z}$ was discretized. The discretization of Equation (20) using an explicit forward difference in time, a first order backward difference and a second order central difference in space results into

(24) $$\begin{array}{c} s=1,\ldots ,S\\ \forall k \end{array}\;\begin{array}{c} c_{2,f}\left(t_{k+1},\left(s-1\right)\cdot \Delta x\right)\approx c_{2,f,s}^{k+1}=c_{2,f,s}^{k}+\frac{\Delta t}{\Delta x^{2}}\cdot \left[c_{2,f,s-1}^{k}-2c_{2,f,s}^{k}+c_{2,f,s+1}^{k}\right]D_{L}^{k}\\ \;+\frac{\Delta t}{\Delta z_{s}}\cdot \left[c_{2,f,s-1}^{k}\cdot u_{s-1}^{\text{k}}-c_{2,f,s}^{k}\cdot u_{s}^{\text{k}}\right]+\Delta t\cdot source_{2,f,s}^{k} \end{array}$$

#### 3.1.2. Discretization of Advection Term: Version B

In version B, the advective term $-u\cdot \frac{\partial c_{2,f}}{\partial z}-c_{2,f}\cdot \frac{\partial u}{\partial z}$ was discretized instead of $-\frac{\partial \left(c_{2,f}\cdot u\right)}{\partial z}\;$. Thus, the discretization of Equation (20) using an explicit forward difference in time, a first order backward difference and a second order central difference in space is given by

(25) $$\begin{array}{c} s=1,\ldots ,S\\ \forall k \end{array}\;\begin{array}{cc} c_{2,f}(t_{k+1},\left(s-1\right) & \cdot \Delta x)\approx c_{2,f,s}^{k+1}\\ & =c_{2,f,s}^{k}\\ & +\frac{\Delta t}{\Delta x^{2}}\{\left[c_{2,f,s-1}^{k}-2c_{2,f,s}^{k}+c_{2,f,s+1}^{k}\right]D_{L}^{k}\;+c_{2,f,s-1}^{k}\cdot u_{s}^{\text{k}}\\ & \cdot \Delta z_{s}+c_{2,f,s}^{k}\cdot u_{s-1}^{\text{k}}\cdot \Delta z_{s}\;-2\cdot c_{2,f,s}^{k}\cdot u_{s}^{\text{k}}\cdot \Delta z_{s}\}+\Delta t\\ & \cdot {source}_{2,f,s}^{k} \end{array}$$

The corresponding discretized initial and boundary conditions for versions A and B are

(26) $$\begin{array}{cc} \forall s,\;k=1 & c_{2,f,s}^{0}=c_{2,\;f,start} \end{array}$$

(27) $$\begin{array}{cc} s=0,\;\forall k & 0=\;-u_{0}^{k}\cdot c_{2,f,0}^{k}+D_{L}^{k}\cdot \frac{c_{2,f,1}^{k}-c_{2,f,0}^{k}}{\Delta x}c_{2,f,0}^{k}=c_{2,f,1}^{k}\cdot \frac{D_{L}^{k}}{D_{L}^{k}+u_{0}^{k}\cdot \Delta x} \end{array}$$

(28) $$\begin{array}{cc} s=S+1,\forall k & c_{2,f,S+1}^{k}=c_{2,f,S}^{k}. \end{array}$$

Equations (24) and (25) can be rearranged to a linear system (Equations (29)–(33)) using the discretized boundary conditions (Equations (27) and (28)) and corresponding cell sizes $\Delta z_{s}$ (Equation (23)).

(29) $$\begin{array}{cc} \forall s,\forall k & c_{2,f}^{k+1}=\;c_{2,f}^{k}-\frac{\Delta t}{\Delta x}\cdot \left(A_{FDM}^{k}\cdot c_{2,f}^{k}-b_{FDM}^{k}\right) \end{array}$$

(30) $$\begin{array}{c} s=1,\ldots ,S\\ \forall k \end{array}\;c_{2,f}^{k}=\left(\begin{array}{c} c_{2,f,1}^{k}\\ \vdots \\ c_{2,f,s}^{k}\\ \vdots \\ c_{2,f,S}^{k} \end{array}\right)$$

∀s,∀k Version A:    $A_{FDM}^{k}=\;\left(\begin{array}{cccccc} \frac{D_{L}^{k}}{D_{L}^{k}+u_{0}^{k}\cdot \Delta x}\cdot (-\frac{D_{L}^{k}}{\Delta x}-2\cdot u_{0}^{k})+\frac{2\cdot D_{L}^{k}}{\Delta x}+2\cdot u_{1}^{k} & -\frac{D_{L}^{k}}{\Delta x} & 0 & \cdots & 0 & 0\\ & & \vdots & & & \\ & & a_{FDM,s}^{T} & & & \\ & & \vdots & & & \\ 0 & 0 & \cdots & 0 & -\frac{D_{L}^{k}}{\Delta x}-2\cdot u_{S-1}^{k} & \frac{D_{L}^{k}}{\Delta x}+2\cdot u_{S}^{k} \end{array}\right)$(31) Version B:       $A_{FDM}^{k}=\;\left(\begin{array}{cccccc} \frac{D_{L}^{k}}{D_{L}^{k}+u_{0}^{k}\cdot \Delta x}\cdot (-\frac{D_{L}^{k}}{\Delta x}-2\cdot u_{1}^{k})+\frac{2\cdot D_{L}^{k}}{\Delta x}+4\cdot u_{1}^{k}-2\cdot u_{0}^{k} & -\frac{D_{L}^{k}}{\Delta x} & 0 & \cdots & 0 & 0\\ & & \vdots & & & \\ & & a_{FDM,s}^{T} & & & \\ & & \vdots & & & \\ 0 & 0 & \cdots & 0 & -\frac{D_{L}^{k}}{\Delta x}-2\cdot u_{S}^{k} & \frac{D_{L}^{k}}{\Delta x}+4\cdot u_{S}^{k}-2\cdot u_{S-1}^{k} \end{array}\right)$
$\begin{array}{c} s=2,\ldots ,S-1\\ \forall k \end{array}$Where $a_{FDM,s}$ is nonzero only for the indices s−1,s,s+1 and given by
Version A:   $a_{FDM,s}=\;\left(\begin{array}{c} 0\\ \vdots \\ -\frac{D_{L}^{k}}{\Delta x}-u_{s-1}^{k}\\ \frac{2\cdot D_{L}^{k}}{\Delta x}+u_{s}^{k}\\ -\frac{D_{L}^{k}}{\Delta x}\\ \vdots \\ 0 \end{array}\right)$Version B:      $a_{FDM,s}=\;\left(\begin{array}{c} 0\\ \vdots \\ -\frac{D_{L}^{k}}{\Delta x}-u_{s}^{k}\\ \frac{2\cdot D_{L}^{k}}{\Delta x}+2\cdot u_{s}^{k}-u_{s-1}^{k}\\ -\frac{D_{L}^{k}}{\Delta x}\\ \vdots \\ 0 \end{array}\right)$(32)

(33) $$\forall s,\forall k\;b_{FDM}^{k}=\;\left(\begin{array}{c} source_{2,f,1}^{k}\cdot \Delta x\\ \vdots \\ source_{2,f,s}^{k}\cdot \Delta x\\ \vdots \\ source_{2,f,S}^{k}\cdot \Delta x \end{array}\right)\;$$

### 3.2. Solving the Diffusion-Advection Equation Using the Finite Volume Method

Under certain conditions, finite volume methods conserve mass discretely and thus are suitable here. In the FVM, the ethanol flux $f_{2}$ (Equation (9)) is evaluated at the cell interfaces $x_{s+\frac{1}{2}}$ and the ethanol concentration $c_{2,f}$ at the nodes $x_{s}$ (Figure 2, Equation (35)). To discretize the boundary conditions appropriately (cf. Equations (42)–(44)), the first discretization node was placed at $z=x_{1}$ and the last node at z=L . Thus, the bottom boundary volume element was halved (Figure 2):

(34) $$\;\Delta z_{s}:=\{\begin{array}{c} \Delta z=\Delta x\;\\ \frac{\Delta z}{2}=\frac{\Delta x}{2}\; \end{array}\begin{array}{c} s=1,\ldots ,S-1\\ s=S\; \end{array}\;.$$

The grid definition of the FVM leads to a slightly different grid compared to the FDMs (including different $\Delta z_{s}$) using the same number of nodes S. This is important for the subsequent comparison of both methods.

To solve Equation (21), we adapted the time-invariant complete flux scheme version developed by Farrell and Linke [11], which is based on Voronoï meshes [13]. They considered a numerical scheme for stationary problems, which we extended to time-dependent problems via integration: Equation (21) was integrated on a discrete cell (Figure 2) at time k yielding Equation (35). Here $f_{2,s+\frac{1}{2}}^{k}$ describes the molar flux which crosses the interface between two neighboring cells. It can be divided into a homogeneous part and an inhomogeneous part (Equation (36)), which were calculated using Equation (37) to Equation (41) according to Farrell and Linke [11]. Their idea is based on complete flux schemes [14,15,16]. Thus, we used the supercritical drying model of gel particles in a packed bed as application example for the complete flux scheme developed by Farrell and Linke [11].

(35) $$\begin{array}{c} s=1,\ldots ,S\\ \forall k \end{array}\;\begin{array}{c} c_{2,f}\left(t_{k+1},\Delta z_{s}\cdot \left(s-0.5\right)\right)\approx c_{2,f,s}^{k+1}\\ \;=\;c_{2,f,s}^{k}-\frac{\Delta t}{\Delta z_{s}}\cdot \left(f_{2,s+\frac{1}{2}}^{k}-f_{2,s-\frac{1}{2}}^{k}\right)+\Delta t\cdot source_{2,f,s}^{k} \end{array}$$

(36) $$\begin{array}{cc} s=1,\ldots ,S\forall k & f_{2,s+\frac{1}{2}}^{k}=\;f_{2,s+\frac{1}{2}}^{k,homogeneous}+f_{2,s+\frac{1}{2}}^{k,inhomogeneous} \end{array}$$

(37) $$\begin{array}{cc} s=1,\ldots ,S\forall k & f_{2,s+\frac{1}{2}}^{k,homogeneous}=-\frac{D_{L}^{k}}{\Delta x}\cdot \left\{B\left(P_{s+\frac{1}{2}}^{k}\right)\cdot c_{2,f,s+1}^{k}-B\left(-P_{s+\frac{1}{2}}^{k}\right)\cdot c_{2,f,s}^{k}\right\} \end{array}$$

(38) $$\begin{array}{cc} s=1,\ldots ,S\forall k & f_{2,s+\frac{1}{2}}^{k,inh.}=-\;\Delta x\cdot \left\{V\left(P_{s+\frac{1}{2}}^{k}\right)\cdot source_{2,f,s+1}^{k}-V\left(-P_{s+\frac{1}{2}}^{k}\right)\cdot source_{2,f,s}^{k}\right\} \end{array}$$

(39) $$\begin{array}{cc} s=1,\ldots ,S\forall k & \mathrm{numerical}\;\mathrm{Pecl}ét\;\mathrm{number}\;P_{s+\frac{1}{2}}^{k}=\frac{u_{s+\frac{1}{2}}^{k}}{D_{L}^{k}}\cdot \;\Delta x \end{array}$$

(40) $$\mathrm{Bernoulli}\;\mathrm{function}\;B\left(x\right):=\frac{x}{e^{x}-1}\;$$

(41) $$\;V\left(x\right):=\frac{e^{\frac{x}{2}}-1-\frac{1}{2}x}{x\left(e^{x}-1\right)}\;$$

The boundary conditions of the FVM (Equations (42)–(44)) were chosen according to Equations (10)–(12). The difference to the FDM (Equations (26)–(28)) is that the boundary conditions are written in terms of ethanol fluxes $f_{2,s}^{k}\;$. Equation (43) implies that no ethanol crosses the upper boundary which is analogous to the Danckwerts’ boundary condition [12]. At the bottom and at the end of the packed bed the ethanol flux is only influenced by the transported ethanol in the bulk fluid (pure convection without any dispersion) (Equation (44)), since the spatial derivative of the ethanol concentration in the bulk fluid $c_{2,f}$ stays zero at z=L (Equation (12)).

(42) $$\begin{array}{cc} \forall s,\;k=1 & c_{2,f,s}^{0}=c_{2,\;f,start} \end{array}$$

(43) $$\begin{array}{cc} s=1,\;\forall k & f_{2,\frac{1}{2}}^{k}=0 \end{array}$$

(44) $$\begin{array}{cc} s=S,\;\forall k & f_{2,S}^{k}=u_{S}^{k}\cdot c_{2,f,S}^{k} \end{array}$$

Equation (35) can be rearranged using Equations (36)–(38), Equations (43) and (44) into a linear system (Equations (45)–(50)) to calculate the unknown ethanol concentration profile $c_{2,f}^{k+1}$ within the bulk fluid along the autoclave height. The division by the vector $\Delta z_{s}$ in Equation (45) is meant component wise (cf. Equations (34) and (47)).

(45) $$\begin{array}{cc} \forall s,\forall k & c_{2,f}^{k+1}=\;c_{2,f}^{k}-\frac{\Delta t}{\Delta z_{s}}\cdot \left(A_{CFS}^{k}\cdot c_{2,f}^{k}-b_{CFS}^{k}\right) \end{array}$$

(46) $$\begin{array}{c} s=1,\ldots ,S\\ \forall k \end{array}\;c_{2,f}^{k}=\left(\begin{array}{c} c_{2,f,1}^{k}\\ \vdots \\ c_{2,f,s}^{k}\\ \vdots \\ c_{2,f,S}^{k} \end{array}\right)\;$$

(47) $$s=1,\ldots ,S\;\;\Delta z_{s}=\left(\begin{array}{c} \Delta z_{1}\\ \vdots \\ \Delta z_{s}\\ \vdots \\ \Delta z_{S} \end{array}\right)$$

(48) $$\forall s,\forall k\;A_{CFS}^{k}=\;\left(\begin{array}{cccccc} \frac{D_{L}^{k}}{\Delta x}\cdot B\left(-P_{\frac{3}{2}}^{k}\right) & -\frac{D_{L}^{k}}{\Delta x}\cdot B\left(P_{\frac{3}{2}}^{k}\right) & 0 & \cdots & 0 & 0\\ & & \vdots & & & \\ & & a_{CFS,s}^{T} & & & \\ & & \vdots & & & \\ 0 & 0 & \cdots & 0 & -\frac{D_{L}^{k}}{\Delta x}\cdot B\left(-P_{S-\frac{1}{2}}^{k}\right) & u_{S}^{k}+\frac{D_{L}^{k}}{\Delta x}\cdot B\left(P_{S-\frac{1}{2}}^{k}\right) \end{array}\right)$$

$\begin{array}{c} s=2,\ldots ,S-1\\ \forall k \end{array}$Where $a_{CFS,s}$ is nonzero only for the indices s−1,s,s+1 and given by$a_{CFS,s}=\;\left(\begin{array}{c} 0\\ \vdots \\ -\frac{D_{L}^{k}}{\Delta x}\cdot B\left(-P_{s-\frac{1}{2}}^{k}\right)\\ \frac{D_{L}^{k}}{\Delta x}\cdot \left[B\left(-P_{s+\frac{1}{2}}^{k}\right)+B\left(P_{s-\frac{1}{2}}^{k}\right)\right]\\ -\frac{D_{L}^{k}}{\Delta x}\cdot B\left(P_{s+\frac{1}{2}}^{k}\right)\\ \vdots \\ 0 \end{array}\right)$(49)

(50) $$\forall s,\forall k\;b_{\mathrm{CFS}}^{k}=\left(\begin{array}{c} \Delta z_{1}\cdot source_{2,f,1}^{k}+\Delta x\cdot \left\{V\left(P_{\frac{3}{2}}^{k}\right)\cdot source_{2,f,2}^{k}-V\left(-P_{\frac{3}{2}}^{k}\right)\cdot source_{2,f,1}^{k}\right\}\\ \vdots \\ \Delta z_{s}\cdot source_{2,f,s}^{k}+\Delta x\cdot \left\{V\left(P_{s+\frac{1}{2}}^{k}\right)\cdot source_{2,f,s+1}^{k}-\left[V\left(-P_{s+\frac{1}{2}}^{k}\right)+V\left(P_{s-\frac{1}{2}}^{k}\right)\right]\;\cdot source_{2,f,s}^{k}+V\left(-P_{s-\frac{1}{2}}^{k}\right)\cdot source_{2,f,s-1}^{k}\right\}\\ \vdots \\ \Delta z_{S}\cdot source_{2,f,S}^{k}\;-\Delta x\cdot \left\{V\left(P_{S-\frac{1}{2}}^{k}\right)\cdot source_{2,f,S}^{k}-V\left(-P_{S-\frac{1}{2}}^{k}\right)\cdot source_{2,f,S-1}^{k}\right\} \end{array}\right)$$

## 4. Results and Discussion

First, we numerically analyze the accuracy of the presented discrete schemes by studying the convergence behavior and the closure of the mole balance. Second, we discuss the computational efficiency of all three methods by examining the condition number as well as the duration of the computation.

The following drying conditions were utilized for all calculations: System pressure P=10 MPa, system temperature T=321 K, gel sphere radius R=3.175 mm, gel porosity $\varepsilon_{g}\;=0.93,$ gel tortuosity $\tau_{g}\;=3.48$, ethanol molar fraction within the gel sphere at the start of the drying $x_{2,g}^{start}=1$, ethanol molar fraction within the gel sphere at the end of the drying (depends on system temperature and defines the drying end) $x_{2,g}^{end}\left(T=321\;K\right)=0.0109$, packed bed porosity ψ=0.4, volume of packed bed $=1.514\cdot 10^{-4}\;m^{3}$, diameter of packed bed $=2.1\cdot 10^{-2}\;m$, mass flow rate of the ethanol–CO_2_ mixture $\dot{m}=0.2003\frac{kg}{s}$. For Figure 9 additional calculations were conducted (see caption of Figure 9).

### 4.1. Accuracy

#### 4.1.1. Convergence Behavior

We would like to know how the number of nodes influences the quality of the calculated solution.

In the following, the convergence behavior is evaluated for two different initial ethanol concentrations in the bulk fluid $c_{2,\;f,start}\;$(Equation (26), Equation (42)), which represent the pressurization using CO_2_:

(a) With excess ethanol: corresponding to $x_{2,f,start}=0.95$;
(b) Without excess ethanol: corresponding to $x_{2,f,start}=0.05\;$.

The analysis of the convergence behavior is based and presented here in form of the ethanol molar fraction instead of the ethanol concentration, since the molar fraction (being proportional to the concentration (Equation (5))) varies from 0 to 1, which allows the reader to easily interpret the results.

**Figure 3:**
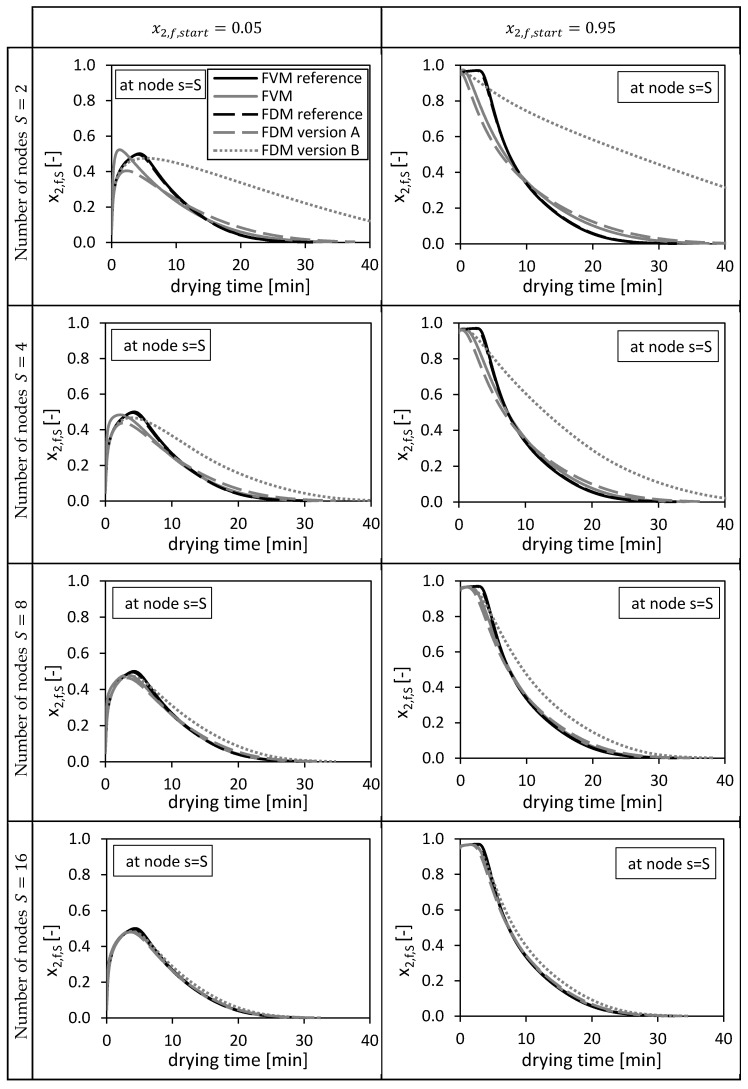

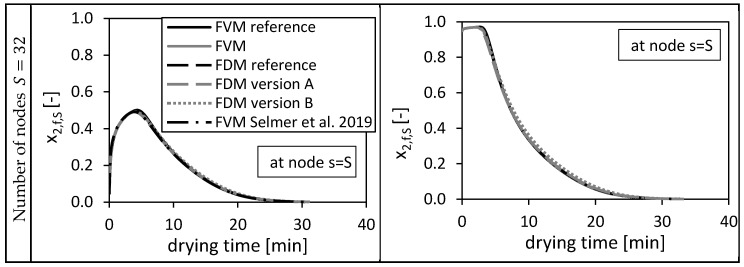
Calculated ethanol mole fraction in the bulk fluid x_2,f,s_ at node s = S, using $x_{2,f}^{\mathrm{start}}$ = 0.05 (left panels) and $x_{2,f}^{\mathrm{start}}$ = 0.95 (right panels). Calculated by the FVM, FDM version A and B and the FVM with the settings used in Selmer et al. 2019 [3] depending on the drying time and the number of nodes S. Calculation settings: N = 26, S_REF_ = 64, Δt = 0.05 s (for FVM, FDM version A and B), Δt = 0.1 s (for FVM Selmer et al. 2019 [3]), S = 20 (for FVM Selmer et al. 2019 [3]).

First, the solutions of the numerical methods are compared at the node s=S (bottom of the packed bed), since this node position is equal for all three methods (Figure 2). Thus, the molar fractions of ethanol in the bulk fluid at the bottom of the packed bed $x_{2,f,S}$ depending on the drying time are shown in Figure 3 for different numbers of grid nodes S (S=2;4;8;16;32). In our case, an analytical solution is missing so that solutions on a fine grid ($S=2^{6}=64$) are used as reference in all cases. The solutions of the FDM calculations are compared with a fine FDM reference solution calculated with FDM version A and S=64 grid nodes in total. The solutions of the FVM calculations are compared with a fine FVM reference solution also calculated with S=64 grid nodes. Two different reference solutions were needed, since especially the first node of the FDM and the FVM differs in its principal position (Figure 2). Both reference solutions presented in form of ethanol molar fraction are additionally shown in Figure 3 and converge to same solutions.

From Figure 3 it is obvious that increasing the total number of nodes S increases the quality of the solutions for all three methods. For S=2 and $x_{2,f,start}=0.05$, the FVM overshoots slightly the reference solutions in the first drying minutes due to a sharp increase of the molar fraction $x_{2,f,S}$ at this position (bottom of the packed) in the beginning of the drying process. The FDM version B prolongs the drying process compared to the reference solutions.

In the previous paper [3], 20 nodes were taken for the same calculation using the presented FVM and $x_{2,f,start}=0.05$ as initial condition. From Figure 3 it is obvious that these 20 nodes (marked as FVM Selmer et al. 2019) were sufficient to calculate a solution being close to the fine reference solution.

**Figure 4:**
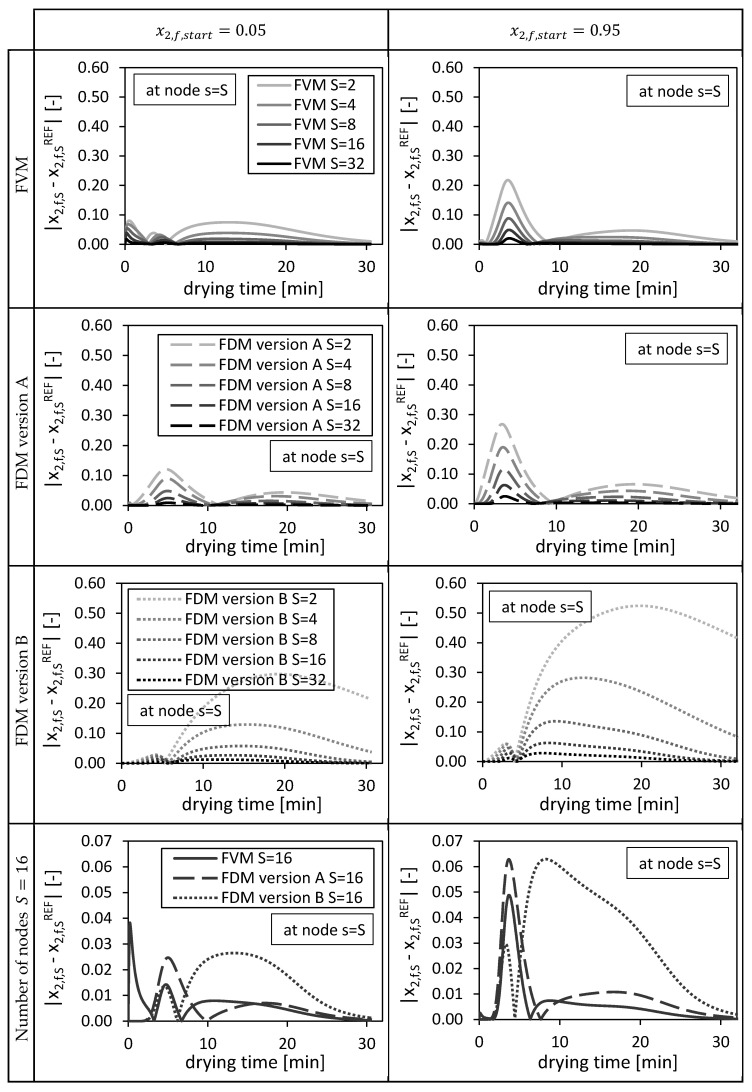
Absolute deviation from reference solution at final node s = S for the FVM, FDM version A and B, using $x_{2,f}^{\mathrm{start}}$ = 0.05 (left panels) and $x_{2,f}^{\mathrm{start}}$ = 0.95 (right panels), depending on the drying time and the number of nodes S. Calculation settings: N = 26, S_REF_ = 64, Δt = 0.05 s (for FVM, FDM version A and B).

The resulting absolute deviations (at position s=S) between the solutions of the investigated numerical methods and their corresponding reference solutions are shown in Figure 4. In general, the deviations of the solutions from the fine reference solutions (Figure 3: in black), restricted to the corresponding coarser meshes, decrease with an increasing number of nodes, indicating convergence (Figure 4). The initial condition of the bulk fluid in the packed bed $x_{2,f,start}$ influences the height as well as the temporal distribution of the deviations (Figure 4). For high temporal changes (Figure 3, $x_{2,f,start}=0.95$) high deviations in case of FDM version A and FVM (Figure 4, $x_{2,f,start}=0.95$) can be observed. Even higher deviations at coarse grids (S=2;4;8) were observed for FDM version B due to a coarser approximation of the convective mass transport in the packed bed (Equation (25)) compared to version A (Equation (24)). The FVM shows smaller deviations compared to the FDMs, except for the usage of S=16 and S=32 nodes in case of $x_{2,f,start}=0.05,$ where relative high deviations could be observed in the first drying minutes (Figure 4). These deviations are induced by the approximated boundary condition of the FVM at the bottom of the packed bed (Equation (44)). The interstitial fluid velocity u and thus the ethanol flow $f_{2}$ at z=L could not be evaluated at the cell interface and had to approximated by using the ethanol concentration $c_{2,f}$ at the final node s=S to estimate the searched ethanol flow (Equation (44)).

**Figure 5:**
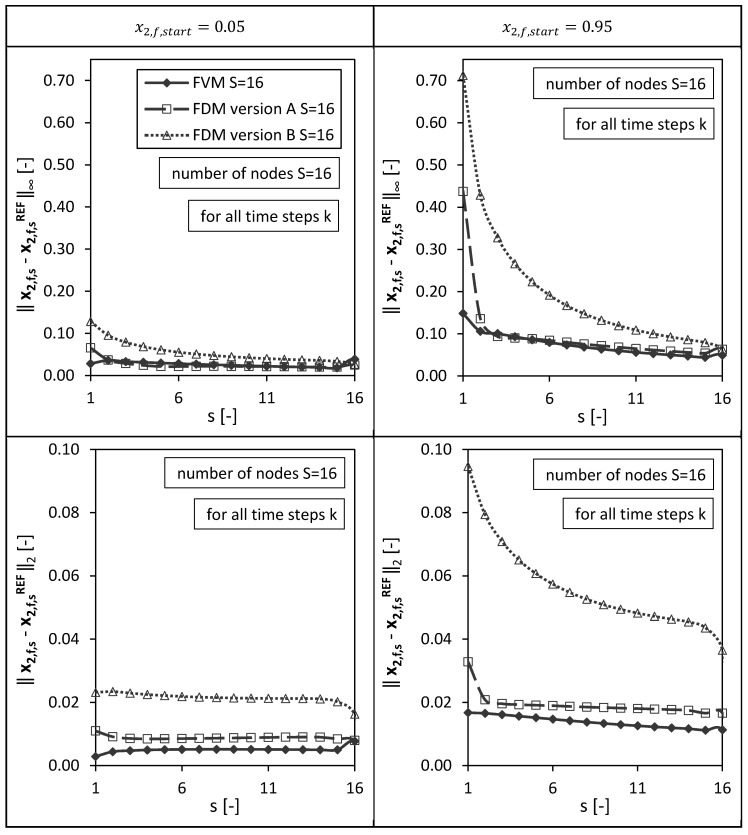
Spatial l_∞_ and l_2_ error distribution calculated for all drying time steps k for the FVM, FDM version A and B using $x_{2,f}^{\mathrm{start}}$ = 0.05 (**left panels**) and $x_{2,f}^{\mathrm{start}}$ = 0.95 (**right panels**). Calculation settings: N = 26, S = 16, S_REF_ = 64, Δt = 0.05 s (for FVM, FDM version A and B).

In a second step, the solutions of the numerical methods were analyzed not only at the final node s=S (bottom of the packed bed) for varying drying times (as presented above), but also for all other nodes ( s=1,…,S ) in order to have a spatial error distribution (Figure 5).

To be able to compare the molar fractions on coarse meshes with the fine reference solution $x_{2,f}^{REF}\;$, the molar fractions on the finer reference mesh were interpolated to the coarse grid. The deviations from the reference solution were calculated for each time step k=1,…,K and for each spatial step s=1,…,S. Thus, the following mole fraction vector was defined to calculate the $l_{\infty }$ and $l_{2}$ errors:

(51) $$\begin{array}{cc} x_{2,f}:={\left(x_{2,f,1},\ldots ,x_{2,f,S}\right)}^{T}\;\mathrm{with}\;x_{2,f,s}={\left(x_{2,f,s}^{1},\ldots ,x_{2,f,s}^{K}\right)}^{T}. & s=1,\ldots ,S \end{array}$$

Figure 5 shows the resulting $l_{\infty }$ and $l_{2}$ errors calculated at each single node s=1,…,16 for all time steps k (as example for a total amount of S=16 nodes). The $l_{\infty }$ error depicts the maximum value of the absolute error, the $l_{2}$ error corresponds to the $l_{2}$ norm of the error.

As observed already in Figure 4, the highest maximum absolute error ($l_{\infty }$ error) at the last node s=S=16 for the case S=16 nodes and $x_{2,f,start}=0.05$ was given by the FVM due to the approximated boundary condition at z=L. Nevertheless, the FVM shows small $l_{\infty }$ errors for all other nodes (similar to FDM version A) for both initial conditions (Figure 5). Overall, the smallest $l_{2}$ errors for all nodes (for all time steps and both initial conditions) were identified for the FVM. Version A of the FDMs shows generally smaller $l_{\infty }$ and $l_{2}$ errors compared to Version B (as already seen for the single node s=S at the bottom of the packed bed (Figure 4)). For both errors, $l_{\infty }$ and $l_{2}$, and all three numerical methods, the errors are generally higher for the initial condition $x_{2,f,start}=0.95$ compared to the case using $x_{2,f,start}=0.05$ due to an almost stepwise change of the molar fraction at the top of the packed bed (s=1) in the beginning of the drying process. The molar fraction $x_{2,f,1}$ changes here at the beginning of the drying process from nearly pure ethanol (component 2) to the autoclave entering pure CO_2_ (component 1). The errors are; therefore, higher at node s=1 compared to the other nodes.

**Figure 6:**
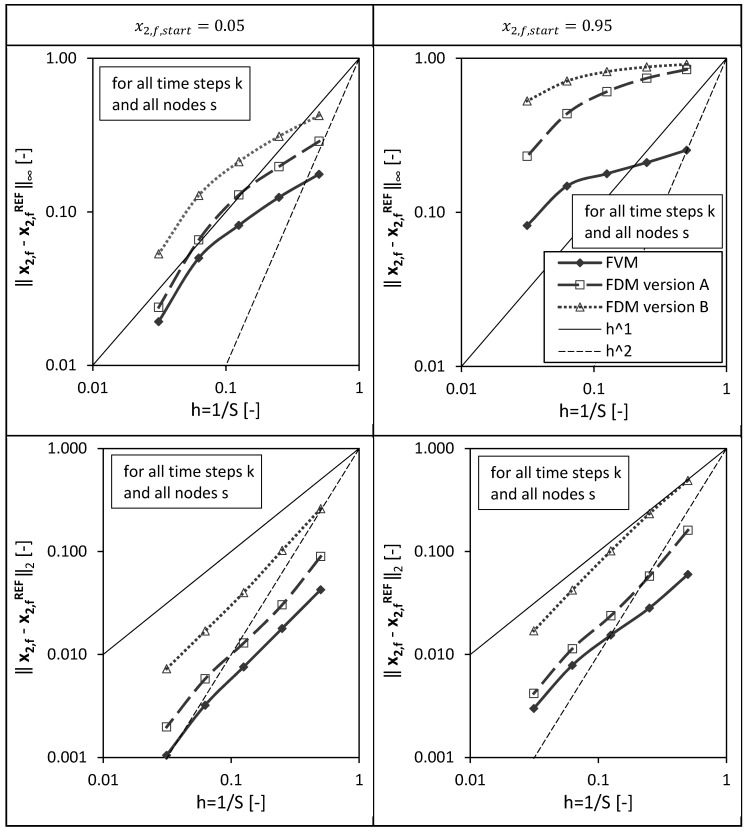
Overall l_∞_ and l_2_ error calculated for all spatial nodes s and for all drying time steps k for the FVM, FDM version A and B using $x_{2,f}^{\mathrm{start}}$ = 0.05 (**left panels**) and $x_{2,f}^{\mathrm{start}}$ = 0.95 (**right panels**) depending on the reciprocal of the total number of nodes S. Calculation settings: N = 26, S_REF_ = 64, Δt = 0.05 s (for FVM, FDM version A and B).

In a third step, the overall errors calculated for all spatial nodes of the packed bed height (s=1,…,S) and for all drying time steps (k=1,…,K) are presented in Figure 6 for different discretizations/grids (S=2;4;8;16;32) and both investigated initial conditions.

It is obvious that the overall errors are higher for the initial condition $x_{2,f,start}=0.95$ compared to the calculations using $x_{2,f,start}=0.05$. For both initial conditions and both errors, $l_{\infty }$ and $l_{2}$, the FVM shows the best convergence behavior, meaning the error decreases the fastest for an increasing total number of grid points S (or smaller grid sizes Δx).

**Figure 7:**
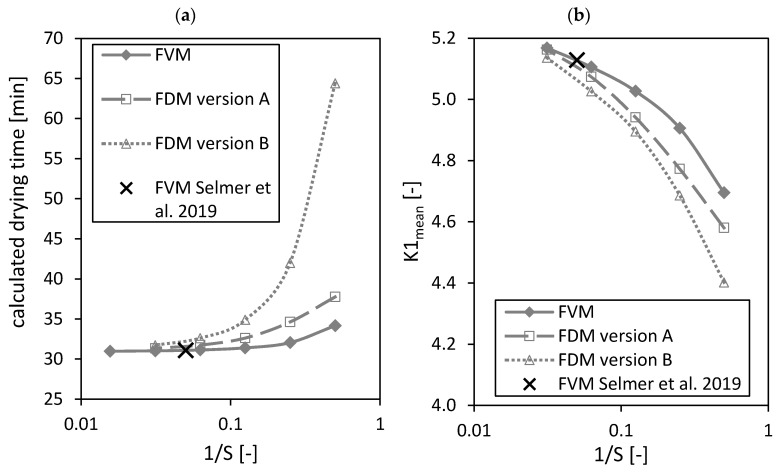
Calculated drying time (**a**) and dimensionless number $K1_{mean}$ (**b**) calculated by the FVM, FDM version A and B and the FVM, with the settings used in Selmer et al. 2019 [3] depending on the reciprocal of the number of nodes S using $x_{2,f}^{\mathrm{start}}$ = 0.05. Calculation settings: N = 26, S_REF_ = 64, Δt = 0.05 s (for FVM, FDM version A and B), Δt = 0.1 s (for FVM Selmer et al. 2019 [3]), S = 20 (for FVM Selmer et al. 2019 [3]).

Next to the above analyzed molar fractions, the calculated drying times as well as the dimensionless number $K1_{mean}$ (both parameters were defined in the previously published paper [3]) are influenced by the total number of grid points S in the region of packed bed (Figure 7). The calculated drying time is the time from start to end of the drying process, whereas the drying process ends when the ethanol molar fraction is less than $x_{2,g}^{\mathrm{end}}\left(T=321\;K\right)=0.0109$ within all gel particles of the packed bed [3]. $x_{2,g}^{\mathrm{end}}$ depends slightly on the system temperature (see previously published paper [3]). The dimensionless number $K1_{mean}$ is a relative measure that shows which mass transport step is the limiting one for the overall drying kinetic, being either diffusion in the gel spheres or mass transport in the bulk fluid. It can be used to find a fast-drying process at low CO_2_ consumption [3].

From Figure 7a it is obvious that a minimum can be reached for the calculated drying time by increasing the number of nodes S. The black crosses in Figure 7 mark the settings of the FVM used in the previously published paper [3]. The calculated drying time from the previously published paper [3] is close to the found minimal value.

An increase of the total number of nodes S leads to an increasing $K1_{mean}$ number (Figure 7b). The result of the previously published paper [3] is again close to the $K1_{mean}$ using the FVM and S=32. Nevertheless, it is unclear if a maximum can be reached by increasing the total number of grid points S further.

#### 4.1.2. Mole Balances

**Figure 8:**
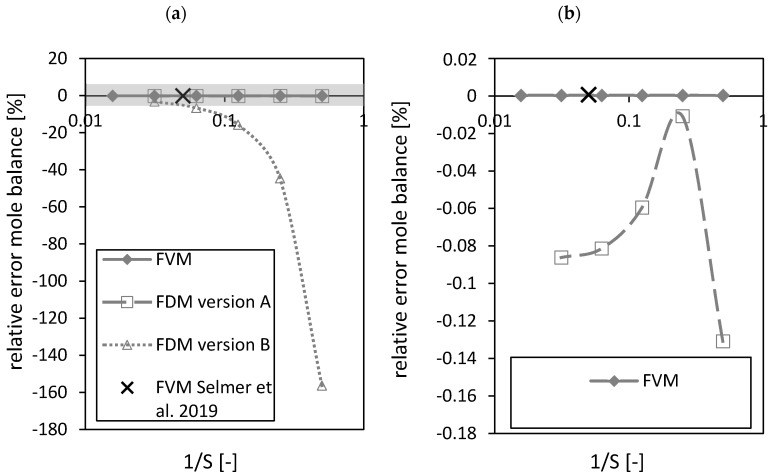
Relative error of the mole balance of the FVM, FDM version A and B and the FVM with the settings used in Selmer et al. 2019 [3] depending on the reciprocal of the number of nodes S using $x_{2,f}^{\mathrm{start}}$ = 0.05 (**a**). Grey area is zoomed in on the right (**b**). Calculation settings: N = 26, S_REF_ = 64, Δt = 0.05 s (for FVM, FDM version A and B), Δt = 0.1 s (for FVM Selmer et al. 2019 [3]), S = 20 (for FVM Selmer et al. 2019 [3]).

During the drying process no chemical conversion occurs and thus the balance in terms of mole fraction of ethanol and carbon dioxide were considered. The closure of the mole balance of ethanol is shown in Figure 8 in the form of the relative error (Equation (52))

(52) $$\;relative\;error\;\left[\%\right]=\left(1-\frac{N_{2,end}}{N_{2,start}}\right)\cdot 100.$$

As expected, the FVM shows the best closure of the mole balance among all discretizations due to its requirement by definition to close the mass balance for each volume element (Figure 8a,b). The relative error of the FDM version A shows a better closure of the mass balance compared to FDM version B (Figure 8a). A maximum (corresponding to an absolute error minimum) was observed for the FDM version A (Figure 8b). The black crosses in Figure 8 mark the settings of the FVM used in the previously published paper [3]. Similar results for the relative errors of the FVM in both studies using different Δt and total number of nodes S were obtained. The relative errors of the FVM (expressed in %) were in the range of 10^−4^.

**Figure 9:**
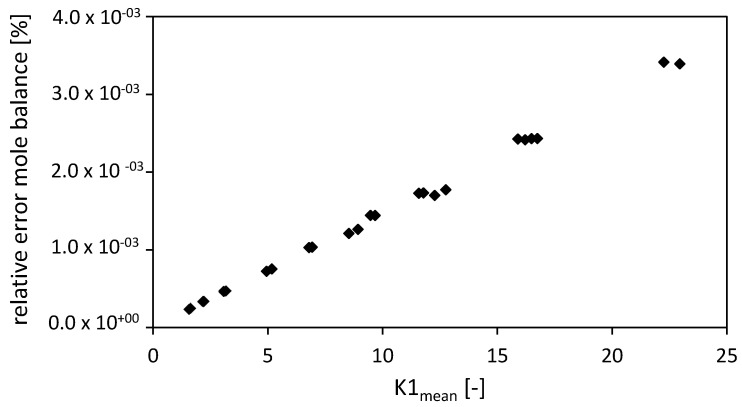
Relative error of the mole balance of the FVM with the settings used in Selmer et al. 2019 [3] depending on the dimensionless number $K1_{mean}$. Drying conditions: P = 9–17 MPa, T = 315–326 K, R = 3.175 mm, gel porosity = 0.93, gel tortuosity = 3.48, $x_{2,g}^{\mathrm{start}}$ = 1, $x_{2,g}^{\mathrm{end}}$ = f(T), bed porosity = 0.4, volume packed bed = 1.514 × 10^−4^ m^3^, diameter packed bed = 2.1 × 10^−2^ m, $x_{2,f}^{\mathrm{start}}$ = 0.05. Calculation settings: N = 26, Δt = 0.1 s, S = 20.

Comparing the closure of the mole balance of the FVM (for fixed Δt and total number of nodes S) depending on the dimensionless number $K1_{mean}$ (Figure 9) shows that the relative error is increasing almost linearly with increasing dimensionless number $K1_{mean}$. An increasing dimensionless number $K1_{mean}$ stands for an increased convective term in the packed bed [3].

For a constant total number of nodes S, an increase of the time step Δt leads to an almost linear increasing relative error (not shown in the diagrams).

Summarizing, the FVM shows the highest approximation quality except for a sharp increase of the molar fraction at the bottom of the bulk fluid (s=S). Version A of the FDM allows for more accurate calculations compared to version B and should be preferred when choosing the FDM.

### 4.2. Efficiency

#### 4.2.1. Condition Numbers

**Figure 10:**
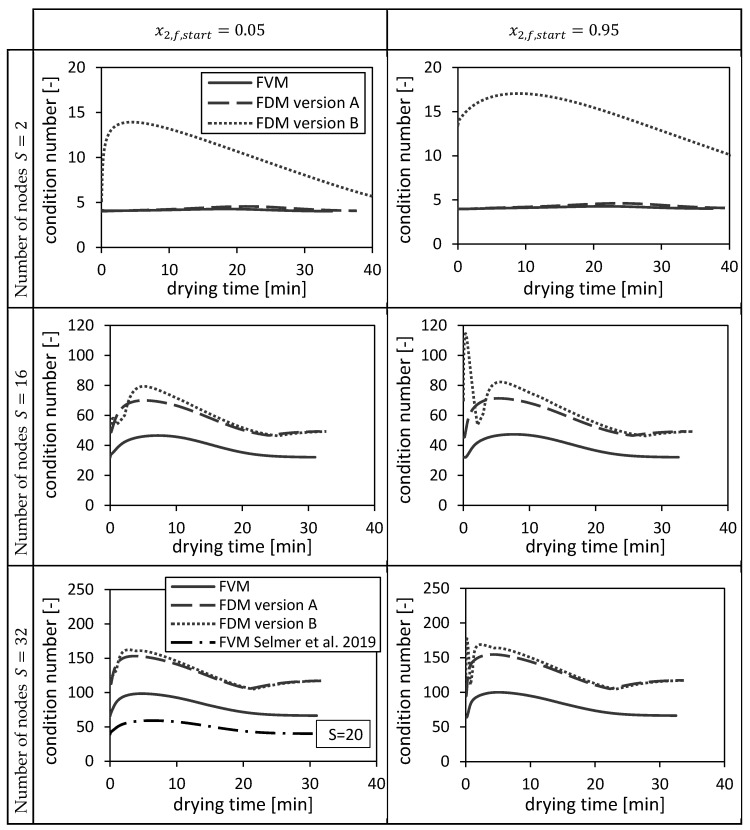
Condition numbers of $A_{CFS}^{k}$ and $A_{FDM}^{k}$ (version A and B) corresponding to the FVM, FDM version A and B and the FVM, with the settings used in Selmer et al. 2019 [3], using $x_{2,f}^{\mathrm{start}}$ = 0.05 (**left panels**) and $x_{2,f}^{\mathrm{start}}$ = 0.95 (**right panels**) depending on the drying time and the number of nodes S. Calculation settings: N = 26, S_REF_ = 64, Δt = 0.05 s (for FVM, FDM version A and B), Δt = 0.1 s (for FVM Selmer et al. 2019 [3]), S = 20 (for FVM Selmer et al. 2019 [3]).

The condition numbers of $A_{CFS}^{k}$ (Equation (48), Equation (49)) and $A_{FDM}^{k}$ (version A and B, Equation (31), Equation (32)) are shown in Figure 10 corresponding to each numerical method for two different initial conditions (cf. Section 4.1.1.). The condition numbers vary with the drying time due to varying dispersion coefficients $D_{L}^{k}$ and interstitial velocities $u_{s}^{k}$ (Equation (31)/Equation (32), Equation (48)/Equation (49)). The condition number of the FVM is the lowest, whereas the condition numbers of the FDMs become closer to each other for increasing number of nodes. The condition number of the FDM version B is up to more than four times higher than the other condition numbers on coarse grids. Additionally, it fluctuates in the beginning of the drying process. The condition number of the FVM used in Selmer et al. 2019 [3] with S=20 nodes is between the condition numbers at S=16 and S=32 of the FVM. The condition number of FDM version B is more sensitive to the initial condition $x_{2,f,start}$ than the condition numbers of the other two methods.

**Figure 11:**
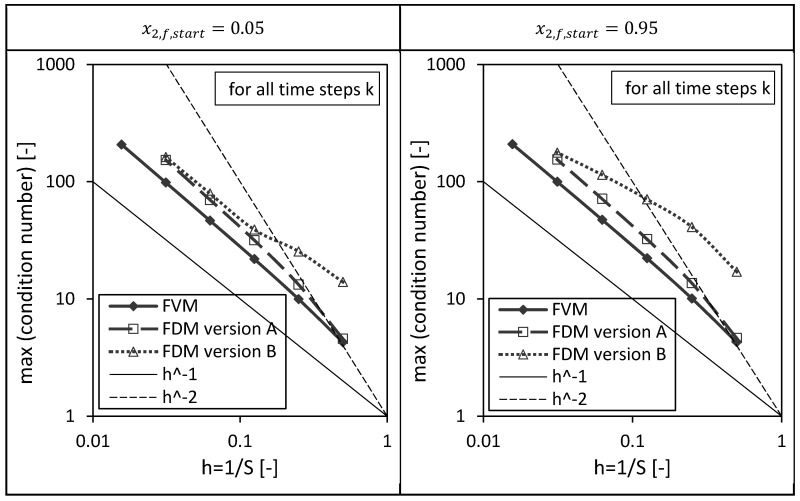
Maximum of the condition numbers of $A_{CFS}^{k}$ and $A_{FDM}^{k}$ (version A and B) corresponding to the FVM, FDM version A and B using $x_{2,f}^{\mathrm{start}}$ = 0.05 (left panel) and $x_{2,f}^{\mathrm{start}}$ = 0.95 (right panel) depending on the reciprocal of the number of nodes S. Calculation settings: N = 26, S_REF_ = 64, Δt = 0.05 s (for FVM, FDM version A and B).

Next to the above presented drying time dependencies of the condition numbers, the corresponding maxima of the condition numbers are shown as a function of the reciprocal of number of nodes S in Figure 11. The previously discussed results can be easily rediscovered here: The FVM has the lowest maximal condition numbers, the maximum condition numbers of all investigated numerical methods increase with increasing number of nodes or decreasing grid sizes and the condition number of the FDM version B is most sensitive with respect to the initial condition.

#### 4.2.2. Computation Time

**Figure 12:**
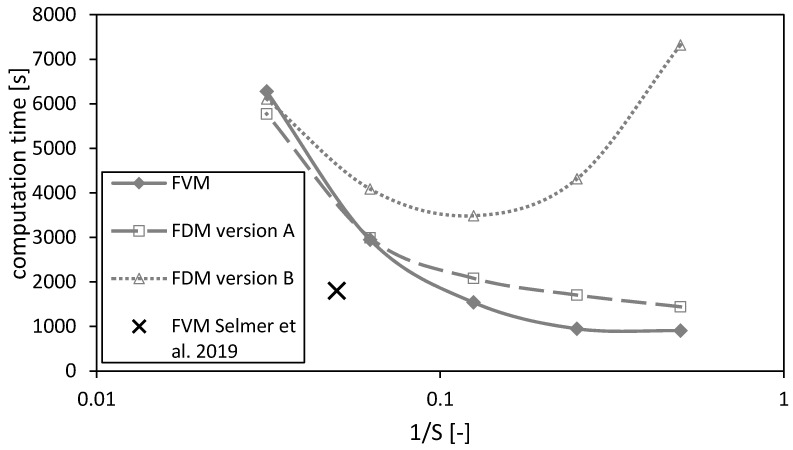
Computation times of the FVM, FDM version A and B and the FVM, with the settings used in Selmer et al. 2019 [3] as function of the reciprocal of the number of nodes S, using $x_{2,f}^{\mathrm{start}}$ = 0.05. Calculation settings: N = 26, S_REF_ = 64, Δt = 0.05 s (for FVM, FDM version A and B), Δt = 0.1 s (for FVM Selmer et al. 2019 [3]), S = 20 (for FVM Selmer et al. 2019 [3]).

The computation time as a function of the reciprocal number of nodes S is shown in Figure 12. In principal, the computation time increases for decreasing mesh sizes (or increasing number of nodes S) for all three methods. The calculations using the FVM take less time than using the FDMs (except on the finest mesh). Additionally, the calculation time increases for the FDM version B for coarse meshes due to an overestimation of the calculated drying time (Figure 7a) and thus longer calculation durations using a fixed time step. Hence, a minimum in the calculation duration of FDM version B can be observed in Figure 12.

The black cross marks the settings of the previously published paper [3]. Its value is smaller than for the calculations of this paper due to a time step which is twice the time step of the calculations made here. Even though the time step is doubled for the calculations in Selmer et al. 2019 [3], the calculation quality is acceptable as shown in the previous sections.

## 5. Conclusions

In this work, we evaluated three different numerical methods, based on finite differences and finite volumes methods, to solve the advection diffusion equation arising in the context of the supercritical drying process of spherical gel particles in a packed bed.

The FVM showed the best closure of the mole balance, best convergence behavior and lowest condition numbers. Therefore, this method is recommended to solve the coupled partial differential equations describing the supercritical drying kinetic in a packed bed developed in Selmer et al. 2018 [2]. A sufficient number of nodes is needed especially for high temporal changes of the molar fractions in the bulk fluid. The reason is that high spatial and/or temporal changes of the molar fractions result into high variations of the physical mixture properties and thus changes all particular mass transfer steps directly or indirectly. Especially the effective diffusion coefficient within the porous gel particle, the mass transfer coefficient and the interstitial fluid velocity within the packed bed are influenced. More detailed information is given in [2,3].

The FDM version A showed good accuracy and computational efficiency. It is easy to implement and should be used with a sufficient number of nodes.

The FDM version B showed the lowest accuracy and efficiency. Thus, it is not recommended to be used.

## Supplementary Materials

The underlying MATLAB codes are available online at https://doi.org/10.6084/m9.figshare.12504863 to provide all necessary details for the implementation of the algorithms presented in the paper.

## Nomenclature

$A_{ac}$	$\left[m^{2}\right]$	Cross-sectional area of the cylindrical autoclave
$a_{CFS,s}$	$\left[\frac{m}{s}\right]$	Row vector in finite volume matrix
$a_{FDM,s}$	$\left[\frac{m}{s}\right]$	Row vector in finite difference matrix
$A_{CFS}$	$\left[\frac{m}{s}\right]$	Finite volume method matrix
$A_{FDM}$	$\left[\frac{m}{s}\right]$	Finite difference method matrix
$A_{p}$	$\left[m^{2}\right]$	Surface area of the spherical particle
$B\left(x\right):=\frac{x}{e^{x}-1}$	[−]	Bernoulli function
$b_{CFS}$	$\left[\frac{mol}{s\cdot m^{2}}\right]$	Right-hand side vector for finite volume method
$b_{FDM}$	$\left[\frac{mol}{s\cdot m^{2}}\right]$	Right-hand side vector for finite difference method
c	$\left[\frac{mol}{m^{3}}\right]$	Concentration
$c_{2,\;f}$	$\left[\frac{mol_{EtOH}}{m^{3}}\right]$	Ethanol concentration in the bulk fluid
$c_{2,\;f}^{k}$	$\left[\frac{mol_{EtOH}}{m^{3}}\right]$	Vector ethanol concentration of the axial bulk fluid elements s
$c_{2,\;g}$	$\left[\frac{mol_{EtOH}}{m^{3}}\right]$	Ethanol concentration within the porous gel particle
$c_{f}$	$\left[\frac{mol_{EtOH}+mol_{CO_{2}}}{m^{3}}\right]$	Mixture concentration in the bulk fluid
$c_{g}$	$\left[\frac{mol_{EtOH}+mol_{CO_{2}}}{m^{3}}\right]$	Mixture concentration within the porous gel particle
D	$\left[\frac{m^{2}}{s}\right]$	Diffusion coefficient
$D_{L}$	$\left[\frac{m^{2}}{s}\right]$	Axial dispersion coefficient in the packed bed
$D_{g}=\frac{\varepsilon_{g}}{\tau_{g}}\cdot D$	$\left[\frac{m^{2}}{s}\right]$	Effective diffusion coefficient within the porous gel particle
f	[−]	Function
$f_{2}$	$\left[\frac{mol}{s\cdot m^{2}}\right]$	Flux of ethanol
h=1/S	[−]	Dimensionless grid size
k	[−]	Time index
K	[−]	Number of time steps
$K1_{mean}$	[−]	Dimensionless number
L	[m]	Length of the packed bed
$l_{2}$	[−]	Euclidean norm
$l_{\infty }$	[−]	Maximum norm
$\dot{m}$	$\left[\frac{kg}{s}\right]$	Mass flowrate of the ethanol–CO_2_ mixture
max	[−]	Maximum
n	[−]	Radial index in gel particle domain
N	[−]	Number of nodes in gel particle domain
$N_{2,end}$	$\left[mol_{EtOH}\right]$	End mole number of ethanol
$N_{2,start}$	$\left[mol_{EtOH}\right]$	Start mole number of ethanol
$N_{p}$	[−]	Number of particles
P	[Pa]	Pressure
P	[−]	Numerical Péclet number
r	[m]	Radial coordinate of the spherical particle
R	[m]	Particle radius
s	[−]	Axial index in autoclave/bulk fluid domain
S	[−]	Number of nodes in autoclave/bulk fluid domain
$source_{2,f}$	$\left[\frac{mol_{EtOH}}{m^{3}\cdot s}\right]$	Ethanol source term within the bulk fluid
t	[s]	Time
T	[K]	Temperature
$u=\frac{U}{\psi }$	$\left[\frac{m}{s}\right]$	Interstitial fluid velocity
U	$\left[\frac{m}{s}\right]$	Superficial fluid velocity
$\dot{V}$	$\left[\frac{m^{3}}{s}\right]$	Volume flow
$V\left(x\right):=\frac{e^{\frac{x}{2}}-1-\frac{1}{2}x}{x\left(e^{x}-1\right)}$	[−]	Function
$V_{f}$	$\left[m^{3}\right]$	Volume bulk fluid
$V_{g,n}$	$\left[m^{3}\right]$	Volume element of spherical gel particle
$x_{2,\;f}$	$\left[\frac{mol_{EtOH}}{mol_{EtOH}+mol_{CO_{2}}}\right]$	Ethanol molar fraction in the bulk fluid
$x_{2,\;f}$	$\left[\frac{mol_{EtOH}}{mol_{EtOH}+mol_{CO_{2}}}\right]$	Vector ethanol molar fraction of the axial bulk fluid elements s and time indices k
$x_{2,\;f}^{REF}$	$\left[\frac{mol_{EtOH}}{mol_{EtOH}+mol_{CO_{2}}}\right]$	Reference vector ethanol molar fraction of the axial bulk fluid elements s and time indices k
$x_{2,\;f,s}$	$\left[\frac{mol_{EtOH}}{mol_{EtOH}+mol_{CO_{2}}}\right]$	Vector ethanol molar fraction of the time indices k
$x_{2,\;g}$	$\left[\frac{mol_{EtOH}}{mol_{EtOH}+mol_{CO_{2}}}\right]$	Ethanol molar fraction within the porous particle
$x_{i}$	$\left[\frac{mol_{i}}{mol_{EtOH}+mol_{CO_{2}}}\right]$	Molar fraction of component i
$x_{s}$	[−]	Node
$x_{s+\frac{1}{2}}$	[−]	Cell interface
z	[m]	Axial coordinate of the autoclave/packed bed
Greek letters		
β	$\left[\frac{m}{s}\right]$	Mass transfer coefficient
Δr	[m]	Distance between neighboring nodes in gel particles
Δt	[s]	Time step
Δx	[m]	Distance between neighboring nodes in autoclave/bulk fluid domain
$\Delta z,\;\Delta z_{s}$	[m]	Length of volume element
$\Delta z_{s}$	[m]	Vector lengths of volume elements
$\varepsilon_{g}$	$\left[\frac{m_{pore\;fluid}^{3}}{m_{particle}^{3}}\right]$	(Aero)gel particle porosity
ψ	$\left[\frac{m_{bulk\;fluid}^{3}}{m_{autoclave}^{3}}\right]$	Porosity of the packed bed (spherical porous particles are here assumed to be nonporous)
$\rho_{f}$	$\left[\frac{kg}{m^{3}}\right]$	Density of ethanol-CO_2_ mixture in the bulk fluid
$\tau_{g}$	[−]	Tortuosity within the porous gel particle
Super and subscripts		
0, start		Start
1		Component carbon dioxide
2		Component ethanol
ac		Autoclave
CFS		Complete flux scheme
$CO_{2}$		Carbon dioxide
end		End
EtOH		Ethanol
f		Bulk fluid
FDM		Finite difference method
FVM		Finite volume method
g,gel		Gel
homogenous		Homogenous
i		Substance component i
inhomogenous, inh.		Inhomogeneous
k		Time index
K		Number of time steps
n		Radial index in gel particle domain
p		Particle
REF		Reference
s		Axial index in autoclave/bulk fluid domain
S		Number of nodes in autoclave/bulk fluid domain
T		Transposed
